# Intercellular adhesion molecule 2 as a novel prospective tumor suppressor induced by ERG promotes ubiquitination-mediated radixin degradation to inhibit gastric cancer tumorigenicity and metastasis

**DOI:** 10.1186/s12967-023-04536-2

**Published:** 2023-09-27

**Authors:** Xiaocheng Tang, Jintuan Huang, Yingming Jiang, Jun Qiu, Tuoyang Li, Weiyao Li, Zijian Chen, Zhenze Huang, Xihu Yu, Tao Yang, Xiang Ji, Rongchang Tan, Li lv, Zuli Yang, Hao Chen

**Affiliations:** 1 Department of Gastrointestinal Surgery Section 2, Department of General Surgery, The Sixth Affiliated Hospital, Sun Yat-Sen University, Guangzhou, 510655 China; 2 Guangdong Provincial Key Laboratory of Colorectal and Pelvic Floor Diseases, The Sixth Affiliated Hospital, Sun Yat-Sen University, Guangzhou, 510655 China

**Keywords:** GC, ICAM2, Transcription factor, Post-translational modifications

## Abstract

**Background:**

Gastric cancer (GC) is a fatal cancer with unclear pathogenesis. In this study, we explored the function and potential mechanisms of intercellular adhesion molecule 2 (ICAM2) in the development and advancement of GC.

**Methods:**

Quantitative real-time polymerase chain reaction (qRT-PCR) and Western blotting were performed to quantify ICAM2 expression in harvested GC tissues and cultured cell lines. Immunohistochemical analyses were conducted on a GC tissue microarray to quantify ICAM2 expression and explore its implication on the prognosis of GC patients. In vitro experiments were carried out to reveal the biological functions of ICAM2 in GC cell lines. Further, in vivo experiments were conducted using xenograft models to assess the impact of ICAM2 on GC development and metastasis. Western blot, immunofluorescence, immunoprecipitation, luciferase assay, chromatin immunoprecipitation, and ubiquitination analysis were employed to investigate the underlying mechanisms.

**Results:**

ICAM2 expression was downregulated in GC, positively correlating with advanced T stage, distant metastasis, advanced clinical stage, vessel invasion, and shorter patient survival time. ICAM2 overexpression suppressed the proliferation, migration, invasion, metastasis of GC cells as well as their ability to form tumors, whereas ICAM2 knockdown yielded opposite results. Erythroblast transformation-specific-related gene (ERG) as a transcription factor promoted the transcription of ICAM2 by binding to the crucial response element localized within its promoter region. Further analysis revealed that ICAM2 reduced radixin (RDX) protein stability and expression. In these cells, ICAM2 bound to the RDX protein to promote the ubiquitination and degradation of RDX via NEDD4 Like E3 Ubiquitin Protein Ligase (NEDD4L), and this post-translational modification resulted in the inhibition of GC.

**Conclusions:**

In summary, this study demonstrates that ICAM2, which is induced by ERG, suppresses GC progression by enhancing the ubiquitination and degradation of RDX in a NEDD4L-dependent manner. Therefore, these results suggest that ICAM2 is a potential prognostic marker and a therapeutic target for GC.

**Supplementary Information:**

The online version contains supplementary material available at 10.1186/s12967-023-04536-2.

## Background

GC is a fatal malignancy, accounting for 5.6% of all human cancers and 7.7% of cancer-related deaths globally, as reported by the World Health Organization in 2020 [1]. Despite recent advancements in the screening and treatment strategies for GC, the prognosis for advanced cases is poor, primarily due to our limited understanding of the molecular mechanisms regulating the progression of this cancer [2–4]. Therefore, there is a need to identify disease markers to improve early detection and treatment of tumors and improve the survival of patients.

ICAM2, a transmembrane glycoprotein, regulates lymphocyte recirculation by inhibiting lymphocyte function-associated antigen-1-dependent cell adhesion and immune surveillance and response by mediating cellular interactions [5–10]. For instance, adenovirus-mediated transfection of ICAM2 was found to improve the adhesion and activation of natural killer cells, resulting in the reduction of peritoneal metastases of GC [11]. In recent studies, ICAM2 was found to be downregulated in specific tumors. This downregulation was associated with malignant characteristics such as, radioresistance, angiogenesis, tumor cell motility, and metastatic potential [12–15]. For example, in oral squamous cell carcinoma cells, exogenous overexpression of ICAM2 significantly reduced cancer cell motility and invasion by inhibiting ERK expression through a mechanism mediated by p53 [16]. In neuroblastoma cells, ICAM2 overexpression may indicate a favorable tumor stage or histology, and the interaction of actin/a-actinin/ICAM-2 forms a complete actin-linker protein-membrane complex that suppresses tumor cell motility in vitro and restricts the metastatic potential of cancer cells in vivo [17]. So far, the function of ICAM2 in gastric cancer (GC) development and its associated pathomechanism is not well understood.

RDX belongs to the ezrin-RDX-moesin family and modulates diverse cellular processes, including differentiation, proliferation, adhesion, maintenance of cell shape, cell motility, and the cross-linking of membranes with cytoskeletal structures [18–21]. Despite the considerable research undertaken on ezrin and moesin, the precise role of RDX in tumor development has not been revealed. Previous studies showed that RDX was overexpressed and promoted tumor malignancy in several types of solid tumors [22]. For example, studies have shown that RDX suppression using RNA interference inhibits the proliferation of human pancreatic cancer cells. This inhibition is due to the upregulation of E-cadherin and thrombospondin-1 [23]. Elsewhere, Zhu et al. demonstrated that RDX knockdown suppressed GC cell invasion and metastasis by improving E-cadherin expression via the nuclear factor kappa B/snail pathway [24]. Paradoxically, miR-31 was reported to promote GC metastasis via negatively regulating RDX [25]. Therefore, further investigations are necessary to explore the precise effects of RDX on GC.

In this study, we found that ICAM2 was significantly downregulated in GC cells and tissues and demonstrated that the decreased expression of ICAM2 was associated with disease progression and predicted poor prognosis. Functional experiments showed that ICAM2 suppressed GC growth and metastasis. Mechanistically, ICAM2 was positively regulated by ERG which resulted in inhibition of GC cells proliferation and motility due to increased NEDD4L-mediated ubiquitination and degradation of RDX. Altogether, these findings demonstrate that ICAM2 is a potential diagnostic and treatment marker for GC patients.

## Methods

### Patients and tissue samples

Samples from both GC and adjacent normal tissues were collected from patients who underwent surgical resection at the Sixth Affiliated Hospital of Sun Yat-sen University in Guangzhou, China between December 2007 and March 2012. Patients who underwent preoperative radiotherapy or chemotherapy, and those diagnosed with other cancer types were excluded. The diagnosis of all specimens was confirmed pathologically. All participants signed written informed consent forms. Immunohistochemical tissue microarrays (TMAs) were constructed using tissue specimens.

### Immunohistochemistry (IHC)

The paraffin-embedded tissue sections were subjected to preparatory steps, including dewaxing, rehydration, and antigen retrieval. 3% H_2_O_2_ and goat serum were separately used to block peroxidase activity and endogenous nonspecific antigens. Subsequently, the sections were subjected to overnight incubation at 4 ℃ in the presence of the primary antibody. After thorough washing with PBS, an HRP-conjugated secondary antibody was applied, and the visualization of the antibody-antigen complex was achieved through the use of a DAB substrate. The final staining scores were determined using an established H-score method [26]. Based on their immunoreactivity scores, the GC patients were divided into two distinct groups, i.e., those with low expression (H-score < 7) and those with high expression (H-score ≥ 7).

### Cell lines and culture

The cells were obtained and cultured as described previously [26, 27].

### Lentivirus production, transfection, and cell infection

Specific silencing and overexpression plasmids (Hanyi Biotechnology) were designed and synthesized to create lentiviruses. As a negative control, empty PCDH and PLKO.1 vectors (Hanyi Biotechnology) were constructed. As previously described [27], lentiviruses were applied to GC cells, and green fluorescent protein-expressing polyclonal cells were selected for further study.

The overexpression plasmid pcDNA3.1-HA-RDX and the empty vector control pcDNA3.1 were both purchased from IGE (IGEbio). Invitrogen Lipofectamine 3000 was used for transfection of GC cells. Cells were subsequently collected within 48-72 h after transfection for further investigation.

### RT-qPCR

For the extraction of total RNA from cells and tissues, the RNA Purification Kit (EZ Bioscience) was used [26]. Reverse transcription and quantification were conducted using the cDNA Reverse Transcription Kit (Applied Biosystems) and the SYBR Green Master Mix Kit (Applied Biosystems) [26].

### Western blotting

To extract cellular proteins, a lysis buffer (Thermo Fisher Scientific) was employed. A BCA protein assay kit (Beyotime) was used to determine the protein concentration. Subsequently, equal amounts of the protein were separated by SDS-PAGE, and then transferred onto polyvinylidene difluoride membranes. These membranes were blocked using 5% skimmed milk and incubated overnight at 4 ℃ with primary antibodies targeting specific proteins, including ICAM2 (Cell Signaling Technology), GAPDH (Proteintech), BAX (ZENBIO), BAD (ZENBIO), BCL2 (Proteintech), E-cadherin (Proteintech), N-cadherin (ZENBIO), Claudin 1 (Proteintech), Snail1 (Proteintech), B-catenin (Abmart), HA (Proteintech), and RDX (ZENBIO). After washing, the membranes were incubated with secondary antibodies (Proteintech) for 1 h at room temperature. They were then visualized using an enhanced chemiluminescence reagent (Meilunbio) and captured using a ChemiDoc Touch imaging system (Bio-Rad).

### Cell proliferation assays

96-well plates were injected with approximately 4 × 10^3^ cells in each well. Incucyte ZOOM (Essen BioScience) photos were captured every 2 h for 72–120 h to determine the well’s cell occupancy region.

### Colony formation assay

About 2 × 10^4^ cells were seeded into each well of 6-well plates and incubated for 7–12 days. The cell medium was replaced every 3 days. After colony incubation period, the cells were washed twice with PBS, and then fixed using 4% paraformaldehyde. Lastly, the cells were subjected to crystal violet staining.

### Migration, invasion, and wound-healing assays

The Transwell (Falcon) assay was employed to study cell migration and invasion. For the invasion assay, Matrigel (60 μl) was introduced to the upper chambers, whereas the migration assay utilized uncoated chambers. In the lower chambers, 700 μl of culture media containing 10% FBS was loaded. A suspension of cells in a serum-free medium was seeded onto the upper chambers. Fixation, staining, photography, and quantification were performed after incubation.

4 × 10^4^ cells were inoculated into a 12-well plate using the Culture-Insert 4 Well (Ibidi). A serum-free RPMI 1640 medium was substituted for the growth medium, and the Incucyte ZOOM was used to monitor the wounds in real time.

### Apoptosis and cell cycle assays

Cells were cultured in a 6-well plate until they reached 80–90% confluence. They were collected and stained with the Annexin V-APC/7-AAD apoptosis kit (MultiSciences) and the cell cycle staining kit (MultiSciences). The data from the apoptosis and cell cycle assays was obtained by using flow cytometry 7 (Beckman Coulter) and subsequently analyzed using CytExpert Version 2.4 (Beckman Coulter) or FlowJo Version 10.0 (BD Biosciences) [26].

### Co-immunoprecipitation (Co-IP) assay

Cells were treated with 500 μl Pierce IP Lysis Buffer (Thermo Fisher Scientific) to obtain cell lysates. The resulting lysates were combined with 50 μl protein A/G agarose beads solution and gently stirred at 4 ℃ for 4 h. Subsequently, specific antibodies or normal immunoglobulin G were added to each tube, and the mixture was incubated overnight at 4 ℃. After washing three times with each 500 μl washing buffer, the immune complexes bound to the beads were thereafter eluted and analyzed through Western blotting.

### Immunofluorescence staining

The cells were fixed using a 4% paraformaldehyde solution, followed by 0.25% Triton X-100 treatment for 15 min to improve permeabilization. Thereafter, a blocking step with 1% BSA was conducted for 30 min to reduce nonspecific binding. Primary antibodies were incubated overnight at a temperature of 4 ℃. The cells were subsequently washed three times to remove unbound antibodies and then stained with Alexa Fluor secondary antibodies (Earthox) and DAPI (Beyotime). Finally, confocal microscopy (Carl Zeiss) was used to capture images.

### Cycloheximide chase and ubiquitination assays

After cells were treated by 100 μg/ml CHX (Selleck) in a gradient time, proteins were extracted, followed by immunoblotting. In the ubiquitination assay, cells were pre-treated with 20 μM MG132 (Selleck) for 4 h before proteins were extracted. The immunoprecipitation (IP) lysis buffer was used for protein extraction, and an anti-RDX antibody was used for IP. Harvested proteins were boiled for 12 min in SDS loading buffer, before immunoblotting.

### ChIP assay

ChIP assays were performed using the Magna ChIP^™^ A/G Chromatin Immunoprecipitation Kit (Merck). Briefly, the cells were fixed with formaldehyde and sonicated, and incubated with the target protein. The cross-linked DNA fragments were then released from the co-precipitated complexes, purified, and resuspended in ddH2O. Through PCR amplification, interactions between ERG and ICAM2 promoter were evaluated. The ChIP-PCR primer targeting the ICAM2 promoter region was (5′ to 3′) ICAM2-site1-F, AAAGCGGAATGGGAGTGG; ICAM2-site1-R, AGTGACGTGCTTCCTGTG.

### Dual-luciferase reporter assays

The luciferase activity of ICAM2-luciferase reporter was measured using the Dual-Luciferase Reporter kit (Meilun). The ICAM2-luciferase reporter plasmid was transfected together with Renilla plasmid into ERG-overexpressing cells and control cells. After 48 h, the luciferase activity was quantified [26].

### In vivo* animal studies*

Female BALB/c nude mice (Guangdong Yaokang Biotechnology) were subcutaneously injected with 1 × 10^6^ ICAM2-overexpressing MGC803 cells or ICAM2-knockdown MKN45 cells via the right flanks. After 3 weeks, mice were euthanized by inhaling CO2, and tumors were harvested and weighed immediately. Based on the following formula, the tumor volume (mm3) was calculated: volume = (length × width^2^)/2.

Approximately 1 × 10^6^ ICAM2-overexpressing MGC803 cells or ICAM2-knockdown MKN45 cells were subcutaneously injected into BALB/c nude mice tail veins to create a lung metastasis model. After 3 months of subcutaneous injection, all mice were euthanized through CO2 inhalation, and then 4% paraformaldehyde was administered to fix the lungs through the main bronchi. After evaluating the metastases, tissue samples were photographed and embedded in paraffin.

### Statistical analysis

Statistical analysis was performed using GraphPad Prism 8.0. Quantitative variables were analyzed using either Student’s t-tests or ANOVA. Data were presented as mean ± standard deviation (SD). To ensure robustness, each experiment was repeated three times. p < 0.05 was considered statistically significant.

## Results

### ICAM2 downregulation in GC cell lines and tissues

**Fig. 1:**
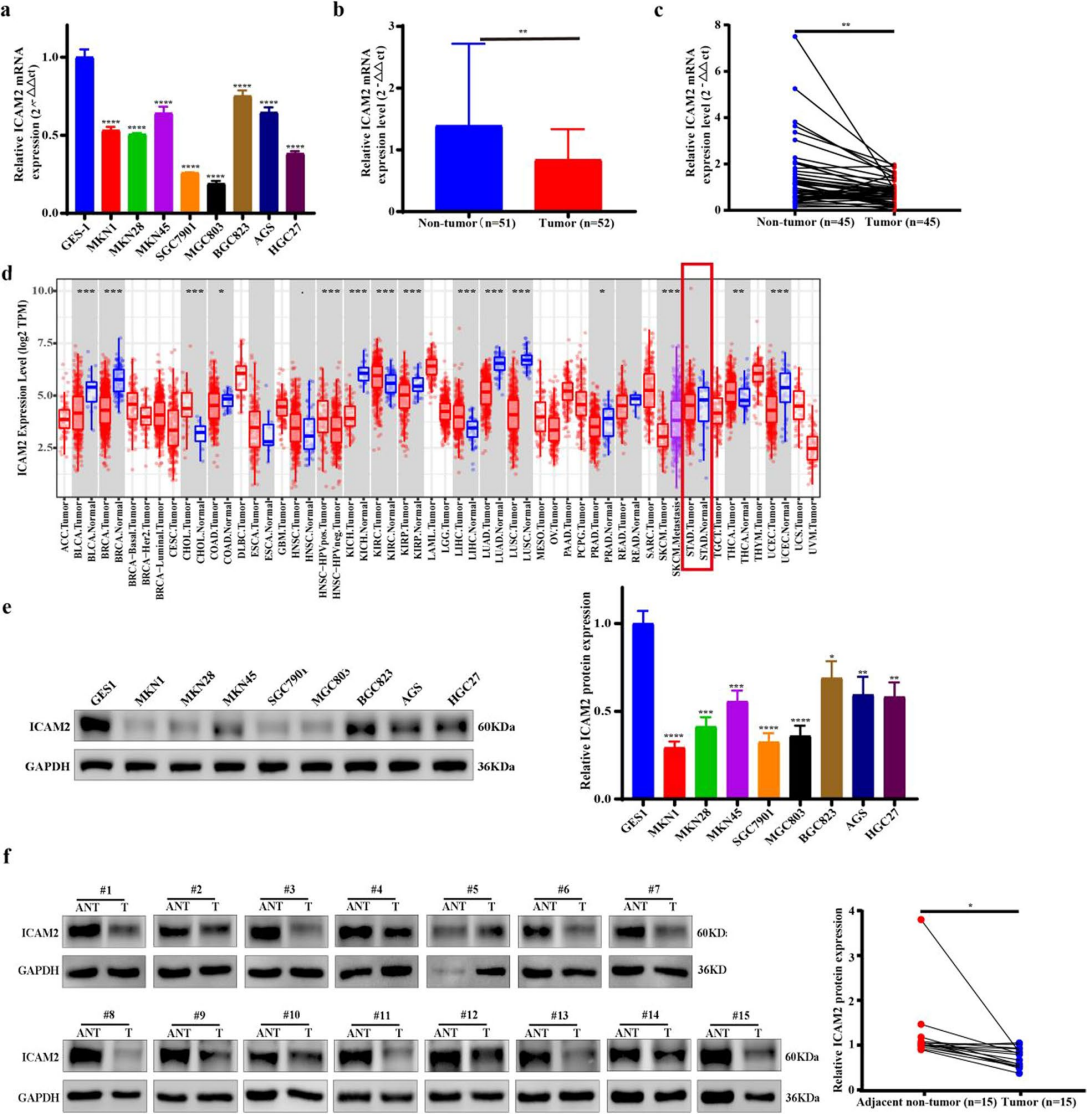
ICAM2 downregulation in GC cell lines and tissues. **a** Comparison of ICAM2 mRNA expression levels in GC cell lines and GES-1. **b**, **c** Comparison of ICAM2 mRNA expression levels in paired and unpaired GC samples. **d** Assessment of ICAM2 transcription levels in the TCGA database across different types of cancer. **e** Evaluation of ICAM2 protein expression in GC cell lines and GES-1. **f** Western blot analysis showing ICAM2 protein expression in 15 paired GC and adjacent nontumor tissues

To explore the role of ICAM2 in GC, we analyzed its expression level in various subtypes of GC cell lines and normal gastric mucosal epithelial cells (GES-1). Our findings revealed that ICAM2 mRNA level was downregulated in GC cell lines compared to GES-1 (Fig. 1a). Similarly, it was downregulated in tumor tissues compared to adjacent normal tissues (Figs. 1b, c). Moreover, analysis of the transcriptome sequencing data obtained from the TCGA database revealed that ICAM2 was downregulated in 10 different tumor types (Fig. 1d). These results provide additional evidence supporting the involvement of ICAM2 in the tumorigenesis of various cancers. To confirm that ICAM2 was also downregulated in GC at the protein expression level, immunoblotting assay was conducted in eight GC cell lines and GES-1. As expected, ICAM2 protein expression was lower in GC cells than in control cells (Fig. 1e). Further tests in 15 tumor tissues and matched adjacent normal tissues (selected randomly from the preceding forty-five cases) showed that ICAM2 protein expression level was decreased in tumor tissues (Fig. 1f). Collectively, these findings demonstrated that ICAM2 was downregulated in GC cell lines and tissue samples, as evidenced by significant reduction at the mRNA and protein levels.

### ICAM2 expression in GC tissues correlates with clinicopathological features and prognosis

**Fig. 2:**
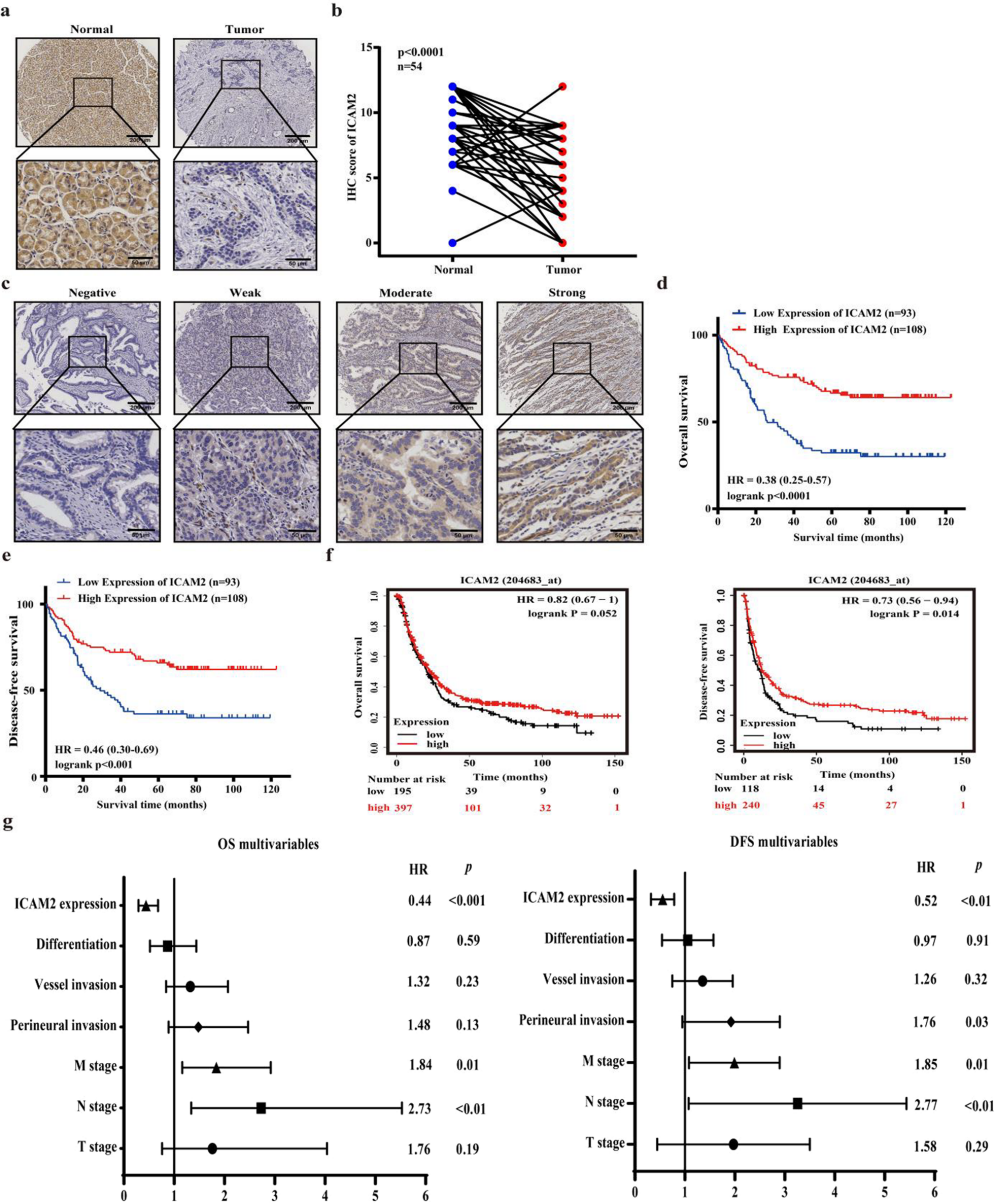
ICAM2 downregulation correlates with poor prognosis of GC. **a** Representative immunohistochemistry images showing ICAM2 expression in GC tissues and their normal controls from cohort 1. **b** A semi-quantitative analysis of ICAM2 IHC staining in paired GC tissues. **c** Representative immunohistochemical staining images for ICAM2 protein in GC in cohort 2. **d**, **e** Kaplan–Meier survival analysis of OS and DFS based on ICAM2 protein levels in GC tissues. **f** Online Kaplan–Meier plotter survival analysis of OS and DFS based on ICAM2 mRNA levels in GC tissues. **g** Multivariable Cox analysis of the clinical prognostic indicators of OS (n = 201) and DFS (n = 201)

Immunohistochemical staining was conducted to explore potential relationships between ICAM2 levels and various clinicopathological features of GC patients. Unlike the matched adjacent non-tumor tissues within cohort 1, there was a significant decrease in ICAM2 expression in GC tissues (Fig. 2a, b). Patients in cohort 2 were categorized into high (*n* = 112) and low (*n* = 99) ICAM2 groups based on the median IHC score (value = 7) (Fig. 2c). Notably, patients in stages III and IV exhibited lower expression levels of ICAM2 compared with those in stages I and II (Table 1). In addition, the relative expression level of ICAM2 was lower in GC patients with T3-4 stage disease compared with those in T1-2 stage. Additionally, patients with distant metastasis or vessel invasion displayed significantly lower ICAM2 expression levels than those without these conditions. Although no statistical significance was detected between ICAM2 expression levels in lymphatic metastasis-positive and negative patients, patients with lymphatic metastasis had lower levels of ICAM2 expression than those without lymphatic metastasis. The Kaplan–Meier survival analysis revealed that patients with low ICAM2 expression had shorter OS and DFS relative to those with high ICAM2 expression (Fig. 2d, e). To validate these findings, online Kaplan–Meier survival curve analysis was conducted which showed that patients with high ICAM2 expression had a markedly longer survival time than those with low ICAM2 expression levels (Fig. 2f). Moreover, the Cox multivariate proportional hazards model demonstrated that decreased ICAM2 expression could independently predict unfavorable OS and DFS in GC patients (Fig. 2g). Collectively, these findings indicated that ICAM2 was a valuable diagnostic and prognostic marker for GC.

**Table 1:** Relationship between ICAM2 Protein Expression Levels and Clinicopathological Characteristics in GC Patients

Variables	Cases	Low ICAM2 (n = 99)	High ICAM2 (n = 112)	p value
Gender				
Male	146	64 (65%)	82 (73%)	0.179
Female	65	35 (35%)	30 (27%)	
Age (years)				
< 60	96	50 (51%)	46 (41%)	0.170
≥ 60	115	49 (49%)	66 (59%)	
pT stage				
T1+T2	43	13 (13%)	30 (27%)	0.014
T3+T4	168	86 (87%)	82 (73%)	
pN stage				
N0	57	21 (21%)	36 (32%)	0.074
N1–N3	154	78 (79%)	76 (68%)	
pM stage				
M0	173	74(75%)	99 (88%)	0.010
M1	38	25 (25%)	13 (12%)	
TNM stage				
I+II	72	24 (24%)	48 (43%)	0.004
III+IV	139	75 (76%)	64 (57%)	
Perineural invasion				
Absent	100	42 (42%)	58 (52%)	0.174
Present	111	57 (58%)	54 (48%)	
Vessel invasion				
Absent	123	49 (49%)	74 (66%)	0.015
Present	88	50 (51%)	38 (34%)	
Differentiation				
Well-moderately	51	18 (18%)	33 (29%)	0.056
Poor	160	81 (82%)	79 (71%)	
Histologic type				
Tubular or papillary	76	28 (28%)	48 (43%)	0.151
Adenocarcinoma		8 (8%)	5 (4%)	
Signet-ring cell carcinoma	13	62 (63%)	58 (52%)	
Mucinous adenocarcinoma	120	1 (1%)	1 (1%)	
Others	2			

### ICAM2 overexpression suppresses GC cell proliferation and tumorigenicity

**Fig. 3:**
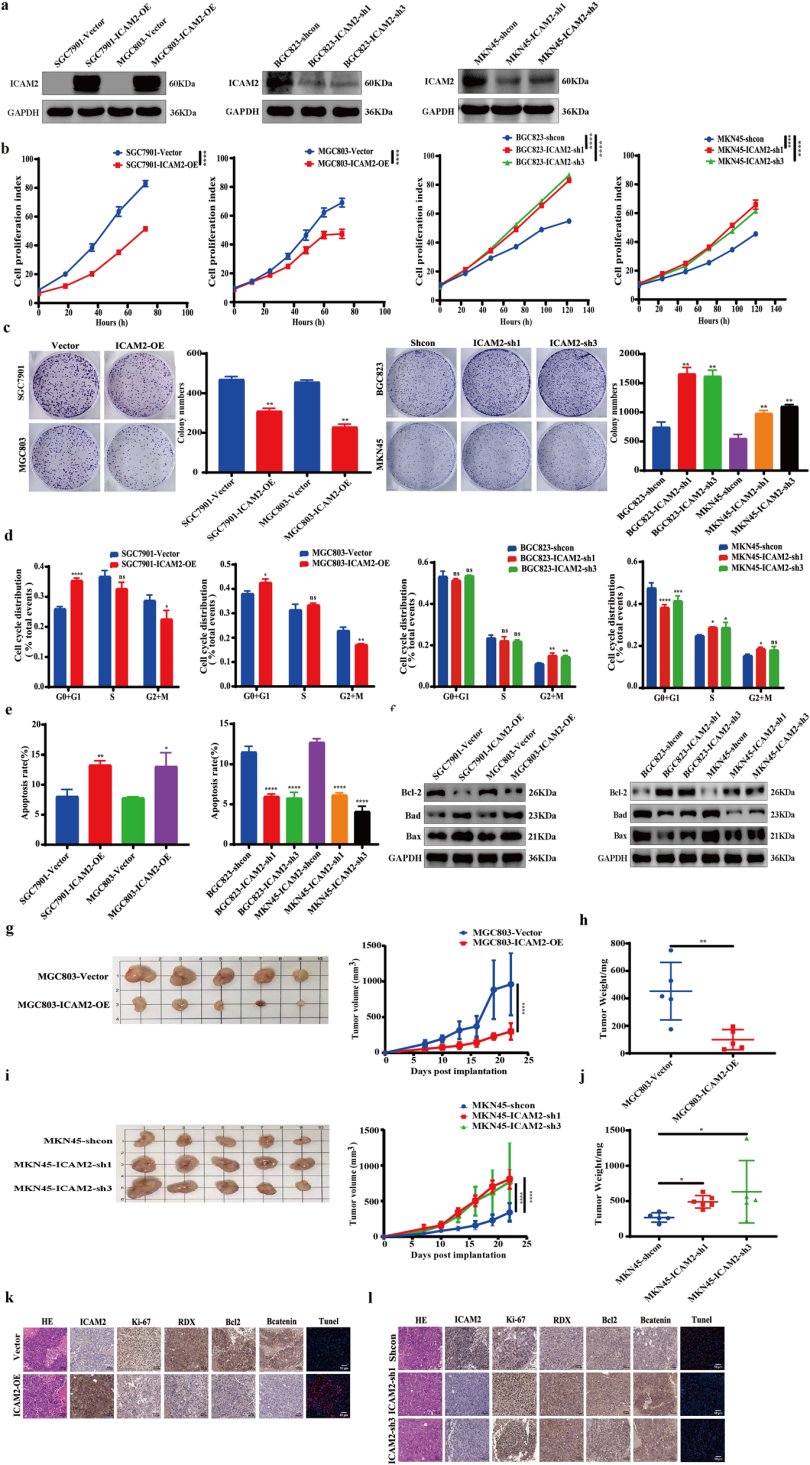
ICAM2 suppresses GC growth in *vitro* and in vivo. **a** Assessment of transfection efficiency by Western blotting after overexpression or knockdown of ICAM2 in GC cells. **b**, **c** Results of cell proliferation assay and colony formation assay demonstrating the effects of ICAM2 overexpression and knockdown on GC cell growth. **d**, **e** Flow cytometry analysis examining the impact of ICAM2 overexpression or knockdown on cell cycle progression and apoptosis in GC cells. **f** Western blotting analysis of apoptosis‐related proteins in ICAM2-overexpressing or knockdown cells. **g**, **h** Growth curve and weight measurements of xenografts in immunodeficient mice with stable expression of vector control or ICAM2. **(i**, **j)** Growth curve and weight measurements of xenografts in immunodeficient mice injected with control or ICAM2-sh cells. **k** H&E and IHC staining images of primary tumor tissue with or without ICAM2 overexpression. **l** H&E and IHC staining images of primary tumor tissue with or without ICAM2 knockdown

To investigate the role of ICAM2 in GC, ICAM2 was overexpressed in SGC7901 and MGC803 cell lines, and silenced in BGC823 and MKN45 cell lines using lentivirus-mediated methods. The success of ICAM2 overexpression and knockdown was verified through RT-PCR and Western blotting assays (Fig. 3a; Additional file 1: Fig. S1a). Subsequently, the cell growth was determined using cell proliferation and colony formation assays. The results showed that ICAM2 overexpression suppressed GC cell proliferation and colony formation, whereas ICAM2 knockdown yielded opposite effects (Fig. 3b, c). Both apoptotic inducers and cell cycle regulators can influence the motility and viability of cells. Analysis of the cell cycle demonstrated that ICAM2 overexpression caused significant cell cycle arrest, characterized by increased cell content in the G0-G1 and decreased cell population in the G2-M phase (Fig. 3d; Additional file 1: Fig. S1b). Conversely, ICAM2 knockdown accelerated cell cycle progression, thereby promoting the proliferation of GC cells (Fig. 3d; Additional file 1: Fig. S1b). Further analysis of flow cytometry data showed that SGC7901 and MGC803 cells transfected with the ICAM2 vector exhibited increased apoptosis rates (Fig. 3e; Additional file 1: Fig. S1c). ICAM2 silencing significantly decreased the apoptosis of BGC823 and MKN45 cells (Fig. 3e; Additional file 1: Fig. S1c). As shown in Fig. 3f, ICAM2 overexpression significantly upregulated the expression of pro-apoptotic proteins BAD and BAX but strongly decreased the expression of anti-apoptotic protein BCL-2 in both SGC7901 and MGC803 cells, whereas ICAM2 knockdown showed opposite results. These findings demonstrate that ICAM2 inhibits the proliferation of GC cells by causing cell cycle arrest and promoting apoptosis.

To investigate whether ICAM2 inhibits GC cell proliferation in vivo, stable ICAM2-overexpressing MGC803 cells and stable ICAM2-knockdown MKN45 cells were used to construct subcutaneous transplantation tumor models in BALB/c-nude mice. The results revealed that ICAM2 overexpression substantially reduced the growth (Fig. 3g) and weight (Fig. 3h) of tumors formed from MGC803 cells. However, ICAM2 knockdown significantly increased the growth (Fig. 3i) and weight (Fig. 3j) of tumors formed by MKN45 cells. This was further confirmed through H&E staining and IHC assays. The results indicated that the xenografts in the ICAM2-overexpressing group exhibited decreased levels of Ki-67 and anti-apoptosis protein BCL-2 expression compared to the control group (Fig. 3k). Conversely, the ICAM2-knockdown group displayed increased levels of Ki-67 and BCL-2 compared to the control group (Fig. 3l). Together, these findings indicated that ICAM2 overexpression inhibited cell proliferation and tumorigenesis.

### *ICAM2 suppresses the metastasis of GC both *in vitro* and *in vivo

**Fig. 4:**
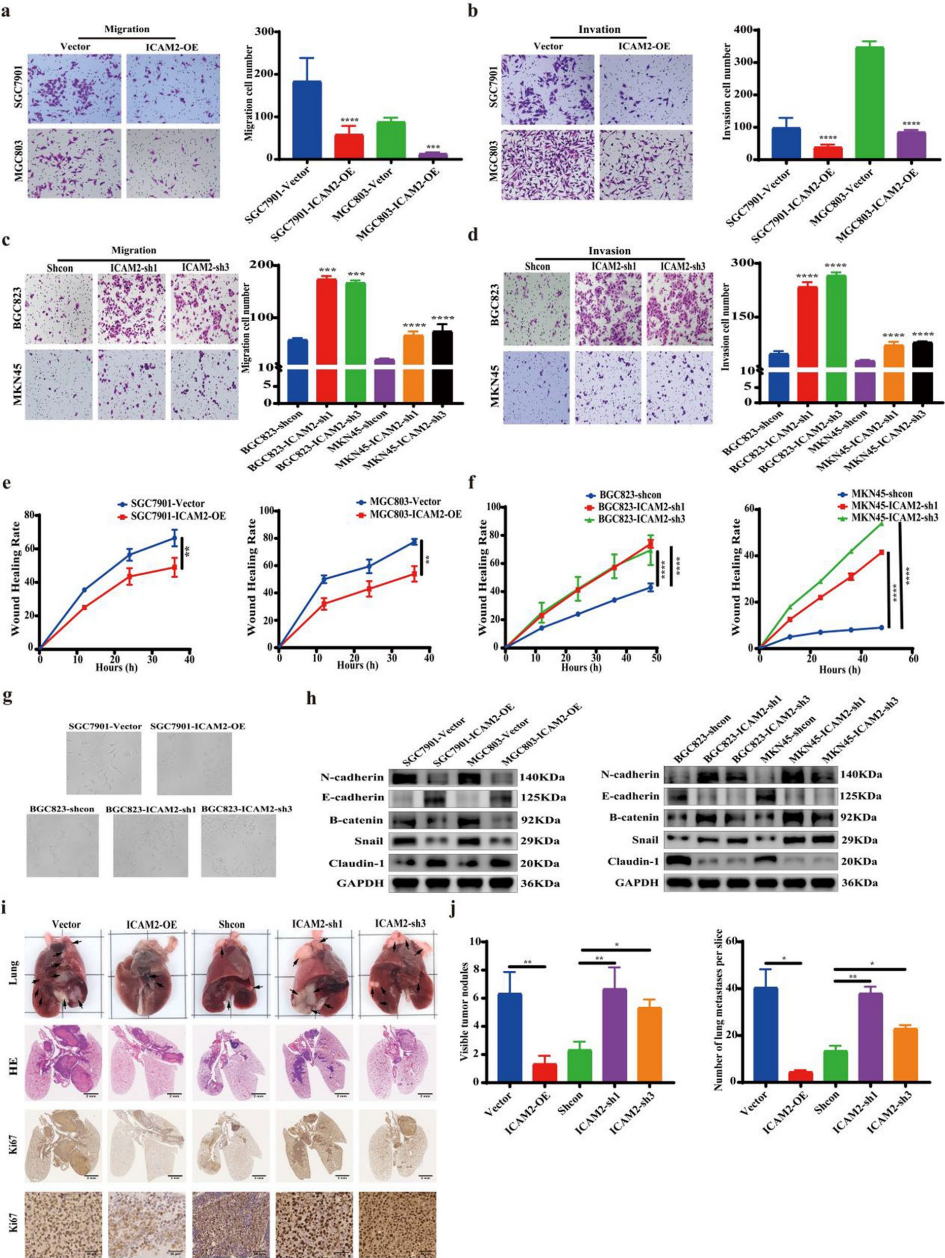
ICAM2 suppresses the GC metastasis. **a–d** Transwell assay investigating the effect of ICAM2 overexpression or knockdown on cell migration and invasion. **e**, **f** Results of the wound healing assay demonstrating the effect of ICAM2 overexpression or knockdown on cell migration. **g** Morphological changes in SGC7901 and BGC823 cells were visualized under an inverted microscope following ICAM2 overexpression or knockdown. **h** Western blot analysis showing the effect of ICAM2 overexpression or knockdown on the expression of EMT-related proteins. **i**, **j** Evaluation of the metastatic potential of cancer cells in vivo by injecting MGC803 (Vector and ICAM2-OE) and MKN45 (Shcon, ICAM2-sh1, and ICAM2-sh3) cells into the tail vein of nude mice. The lungs with metastatic lesions were collected and subjected to histopathological examination using H&E staining and Ki67 staining

We conducted transwell and wound healing assays to examine the effect of ICAM2 on GC metastasis. Consequently, ICAM2 overexpression significantly reduced the number of migration and invasion cells in SGC7901 and MGC803 cells (Fig. 4a, b) and inhibited wound healing (Fig. 4e; Additional file 1: Fig. S1d). Moreover, ICAM2 knockdown promoted the migration (Fig. 4c), invasion (Fig. 4d), and wound healing (Fig. 4f; Additional file 1: Fig. S1d) capacity of MKN45 and BGC823 cells. Moreover, ICAM2-knockdown and overexpressing cells showed distinct cell morphology. Compared with the control cells, ICAM2-silenced cells presented typical epithelial-to-mesenchymal (EMT) morphological features, transformed them from a dense epithelial morphology with marked intercellular junctions to a fibrous morphology with a dispersed spindle-like structure, whereas ICAM2 overexpression showed opposite effects (Fig. 4g). The expression of EMT-related proteins was significantly altered in ICAM2 knockdown GC cells, where the epithelial markers Claudin-1 and E-cadherin were downregulated whereas that of the mesenchymal markers B-catenin, N-cadherin, and Snail1 were increased (Fig. 4h). In contrast, ICAM2 overexpression in SGC7901 and MGC803 cells decreased N-cadherin, B-catenin, and Snail1, and increased E-cadherin and Claudin-1 protein expression compared to control cells (Fig. 4h). These findings suggested that ICAM2 regulated EMT gene expression to suppress GC cell mobility.

A mouse model of lung metastasis was developed to assess the antitumor effects of ICAM2 in vivo. Consistent with the findings in the subcutaneous transplantation tumor model, ICAM2 overexpression significantly decreased lung metastatic lesions originating from MGC803 cells (Fig. 4i, j). Conversely, ICAM2 knockdown increased lung metastatic lesions formed by MKN45 cells (Fig. 4i, j). Similarly, H&E staining confirmed that the ICAM2 overexpression reduced metastatic lesions, whereas the ICAM2 knockdown increased them (Fig. 4i, j). Proliferation was evaluated in resected lung tissues stained with Ki-67, and the result revealed that ICAM2 overexpression impaired proliferation in lung metastases derived from MGC803 cells, whereas ICAM2 knockdown promoted proliferation in those derived from MKN45 cells (Fig. 4i). Collectively, these findings indicated that ICAM2 inhibited the metastatic potential of GC cells.

### ICAM2 binds to RDX and promotes its ubiquitination and degradation

**Fig. 5:**
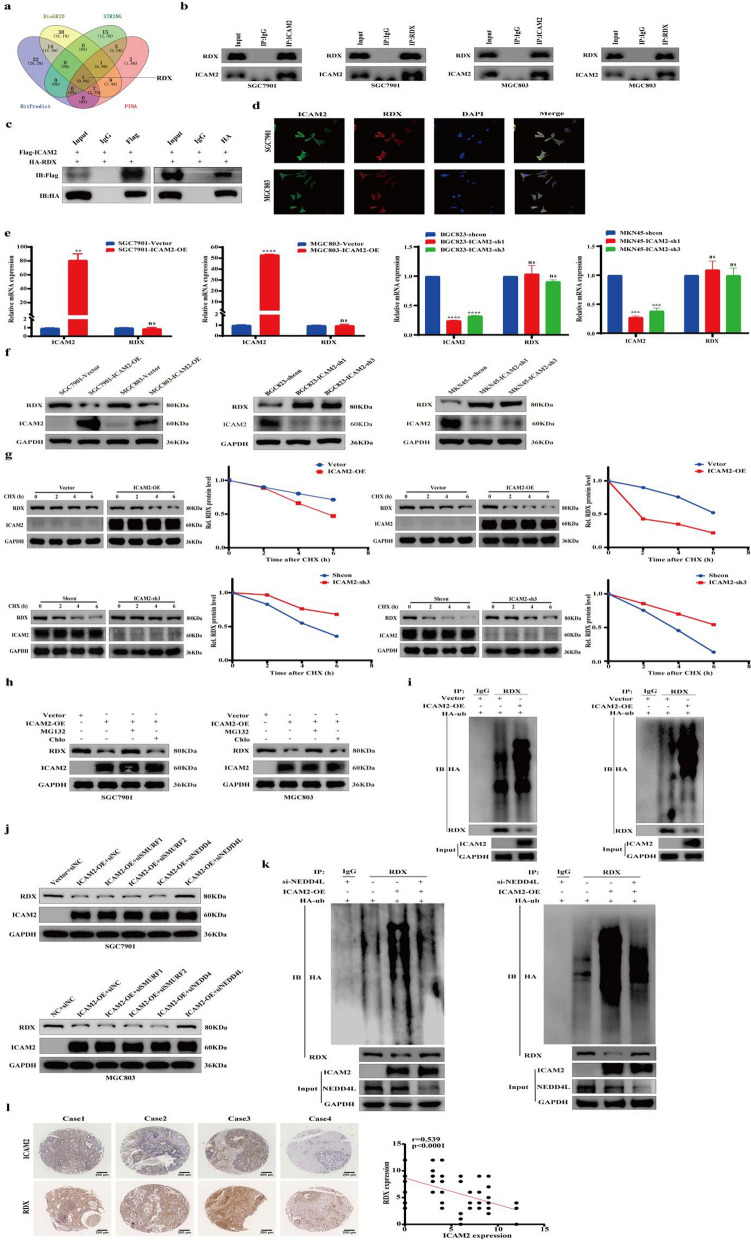
ICAM2 mediates RDX ubiquitination degradation in a NEDD4L-dependent manner. **a** Bioinformatic analysis of potential interacting proteins of ICAM2. **b** Co-IP was conducted to determine the interaction between endogenous ICAM2 and RDX in SGC7901 and MGC803 cells. **c** Co-IP was performed to detect the interaction between exogenous ICAM2 and RDX by co-transfecting Flag-ICAM2 and HA-RDX plasmids into 293 T cells. **d** Immunofluorescence staining examining the colocalization of ICAM2 and RDX in GC cells. **e** Quantification of RDX mRNA expression using RT-PCR following ICAM2 overexpression or knockdown. **f** Western blot analysis of RDX protein expression after ICAM2 overexpression or knockdown. **g** CHX chase assay to explore the half-life of RDX protein in the presence or absence of ICAM2. **h** Evaluation of the stability of RDX protein through chloroquine or MG132 treatment in the control and ICAM2 overexpression groups. **i** RDX ubiquitination level was detected by the Co-IP and Western blotting assays in the ICAM2 overexpression and control groups. **j** Western blot analysis investigating the effects of knockdown of the main E3 ubiquitin ligases on RDX protein levels in ICAM2 overexpression GC cells. **k** Assessment of the ubiquitination level of RDX in GC cells with ICAM2 overexpression following NEDD4L knockdown. **l** Representative images of ICAM2 and RDX immunohistochemically stained in paired GC tissues. Linear regression analysis of ICAM2 and RDX expression

Considering that ICAM2 is involved in tumor development, we explored the mechanisms by which ICAM2 inhibited cell growth and metastasis. Given that the effects of ICAM2 on neuroblastoma cell phenotype are influenced by ICAM2 interaction with the cytoskeletal linker protein, we speculated that ICAM2 may interact directly with potential proteins to regulate the function of GC cells [15]. To identify the proteins that bind to ICAM2, HitPredict, String, PINA, and BioGrid databases were used to screen the potential interacting proteins. It was found that RDX—a cytoskeletal linker protein reported to promote tumor pathogenesis—was the only protein in the intersection (Fig. 5a). Studies have shown that interactions between the cytoskeleton and cell surface adhesion molecules modulate various cell functions such as, proliferation, morphology, and mobility. Therefore, we investigated the relationship between ICAM2 and RDX. First, we analyzed the interaction between ICAM2 and RDX using Co-IP experiments. In line with the results of the database analysis, endogenous RDX was pulled down by the ICAM2 antibody (Fig. 5b). Similar results were also observed when the RDX antibody was employed to immunoprecipitate endogenous ICAM2 (Fig. 5b). Exogenous Co-IP revealed similar results (Fig. 5c). Besides, immunofluorescence assays revealed that ICAM2 was co-localized with RDX in GC cells (Fig. 5d). Since ICAM2 interacts with RDX, we investigated whether ICAM2 modulated RDX expression. It was observed that ICAM2 did not affect the mRNA level of RDX (Fig. 5e). Western blot analysis showed that ICAM2 overexpression reduced RDX protein expression levels, whereas ICAM2 silencing significantly increased RDX protein expression levels (Fig. 5f). This suggested that ICAM2 regulated RDX protein expression at the post-transcriptional level. Further, we used CHX, a protein synthesis inhibitor, to detect the stability of RDX in cells with ICAM2overexpression and knockdown. There was a significant reduction in the half-life of RDX protein in ICAM2-overexpressing cells, whereas ICAM2-knockdown cells exhibited a prolonged half-life of RDX protein compared to the control cells (Fig. 5g). These findings provided compelling evidence that ICAM2 was involved in the regulation of RDX protein stability. In eukaryotes, there are two major pathways for protein degradation, i.e., the proteasome and autophagy-lysosome. To identify the pathway by which ICAM2 promoted RDX protein degradation, SGC7901 and MGC803 cells were separately pretreated with the proteasome inhibitor MG-132 and the autophagy-lysosome inhibitor chloroquine, and the expression level of RDX protein was detected. Significant RDX deposition was observed in ICAM2-overexpressing cell lines after pretreatment with MG132 but not chloroquine, indicating that ICAM2 reduced RDX expression through the proteasomal pathway (Fig. 5h). Given the dominant role of the ubiquitin–proteasome pathway in protein degradation, we sought to determine whether ICAM2 promoted RDX ubiquitination to reduce it in GC cells. RDX was immunoprecipitated using anti-RDX antibody, and its ubiquitination status was determined. As predicted, ICAM2 overexpression increased the level of RDX ubiquitination (Fig. 5i). These results suggested that ICAM2 destabilized RDX by affecting ubiquitination-mediated protein degradation. The UbiBrowser was used to predict E3 ubiquitin ligases of RDX, which showed that SMURF1, SMURF2, NEDD4, and NEDD4L were the main E3 ubiquitin ligases mediating RDX ubiquitination and degradation (Additional file 2: Fig. S2a). Further, these E3 ubiquitin ligases were knocked down by siRNA in ICAM2 overexpression cells and consequently, the inhibitory effect of ICAM2 overexpression on RDX protein levels was abolished only by NEDD4L in SGC7901 and MGC803 cells (Fig. 5j; Additional file 2: Fig. S2b). This indicated that ICAM2 promoted RDX degradation in a NEDD4L-dependent manner. Results of the Co-IP experiments provided additional support to our findings. Specifically, they showed that the depletion of NEDD4L abolished the effect of ICAM2 on RDX ubiquitination and degradation (Fig. 5k). To further confirm the correlation between ICAM2 and RDX in GC tissues, we performed IHC. Results revealed a negative correlation between the expression of ICAM2 and RDX (Fig. 5l). Taken together, these findings provide compelling evidence that ICAM2 improves ubiquitin/proteasome-dependent degradation of the RDX protein in a NEDD4L-dependent manner, thereby decreasing RDX expression levels.

### RDX activation reverses the suppressive effects of ICAM2 overexpression on the proliferation and metastasis of GC

**Fig. 6:**
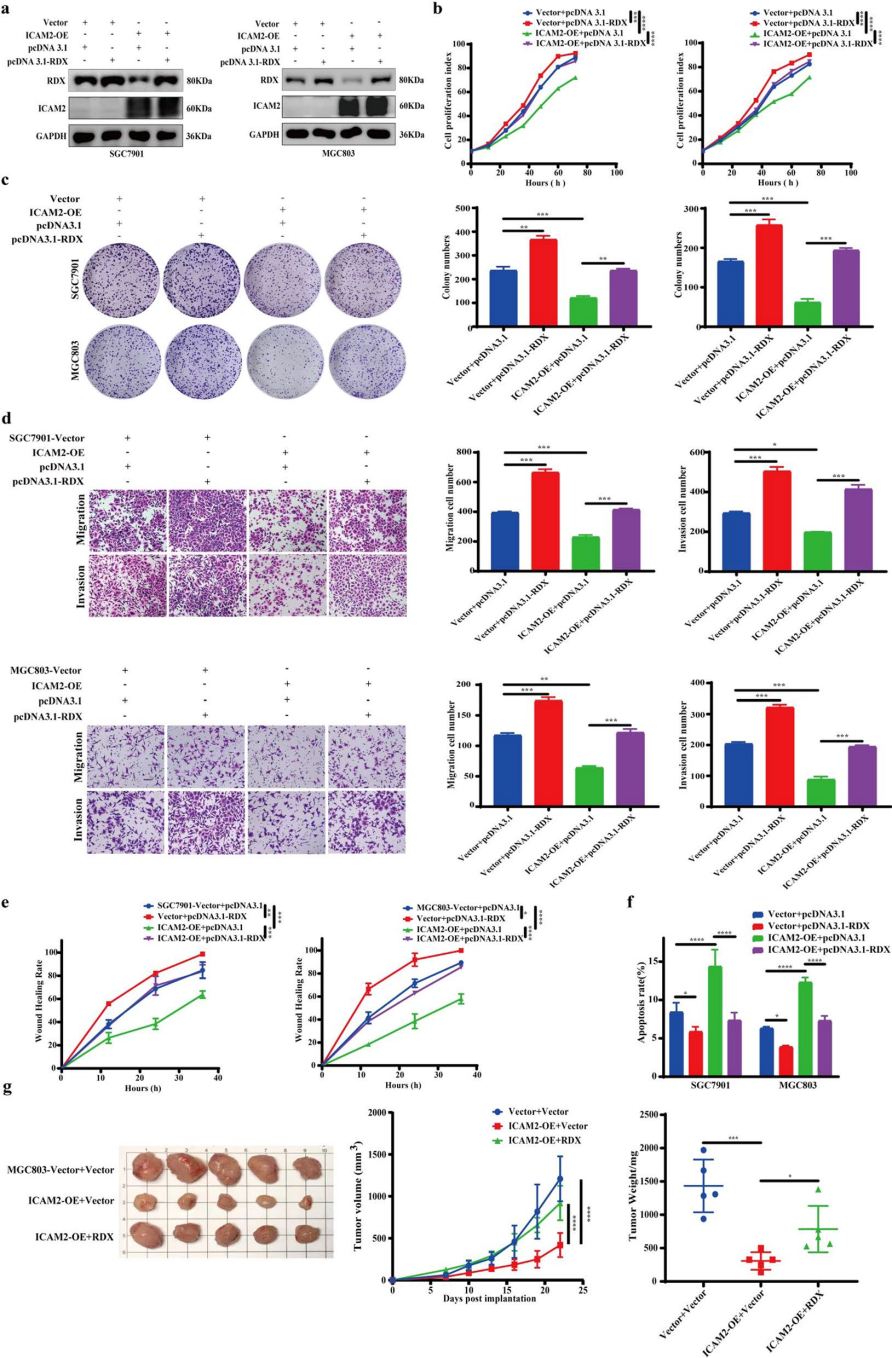
The dependence of ICAM2 inhibitory effects on RDX inactivation. **a** RDX overexpression was validated using Western blotting. **b**, **c** Cell proliferation and colony formation experiments were conducted to assess the proliferation ability of ICAM2‐overexpressing cells in the presence or absence of RDX. **d**, **e** Transwell assay and wound-healing assay were performed to determine the mobility of ICAM2‐overexpressing cells treated with RDX. **f** Flow cytometry was employed to determine the cell apoptosis of ICAM2‐overexpressing cells treated with RDX. **g** Evaluation of the growth curve and weight of xenografts in immunodeficient mice generated by subcutaneous injection of MGC803 cells stably transfected with Control, ICAM2, Vector, or RDX

In further tests, we explored whether RDX mediates the anti-proliferation and anti-metastatic effects of ICAM2. Several rescue experiments were performed to test this hypothesis. Initially, exogenous RDX was introduced into ICAM2-overexpressing cells, and RT-qPCR and Western blot assays were carried to confirm the successful transfection (Fig. 6a; Additional file 3: Fig. S3a). Notably, ICAM2 overexpression suppressed the proliferation and colony formation processes of both SGC7901 and MGC803 cells, whereas exogenous RDX abolished this effect (Fig. 6b, c). RDX overexpression also alleviated the ICAM2 overexpression-induced anti-migration and anti-invasion processes of GC cells (Fig. 6d, e; Additional file 3: Fig. S3b). Additionally, RDX overexpression reversed ICAM2-mediated promotion of apoptosis (Fig. 6f; Additional file 3: Fig. S3c). In xenograft tumor models, Overexpression of RDX in MGC803 cells erased ICAM2-induced growth rate and tumor weight differences (Fig. 6g; Additional file 3: Fig. S3d). These findings suggested that RDX mediated the regulatory function of ICAM2 in GC.

### ERG promotes the transcription of ICAM2 in GC

**Fig. 7:**
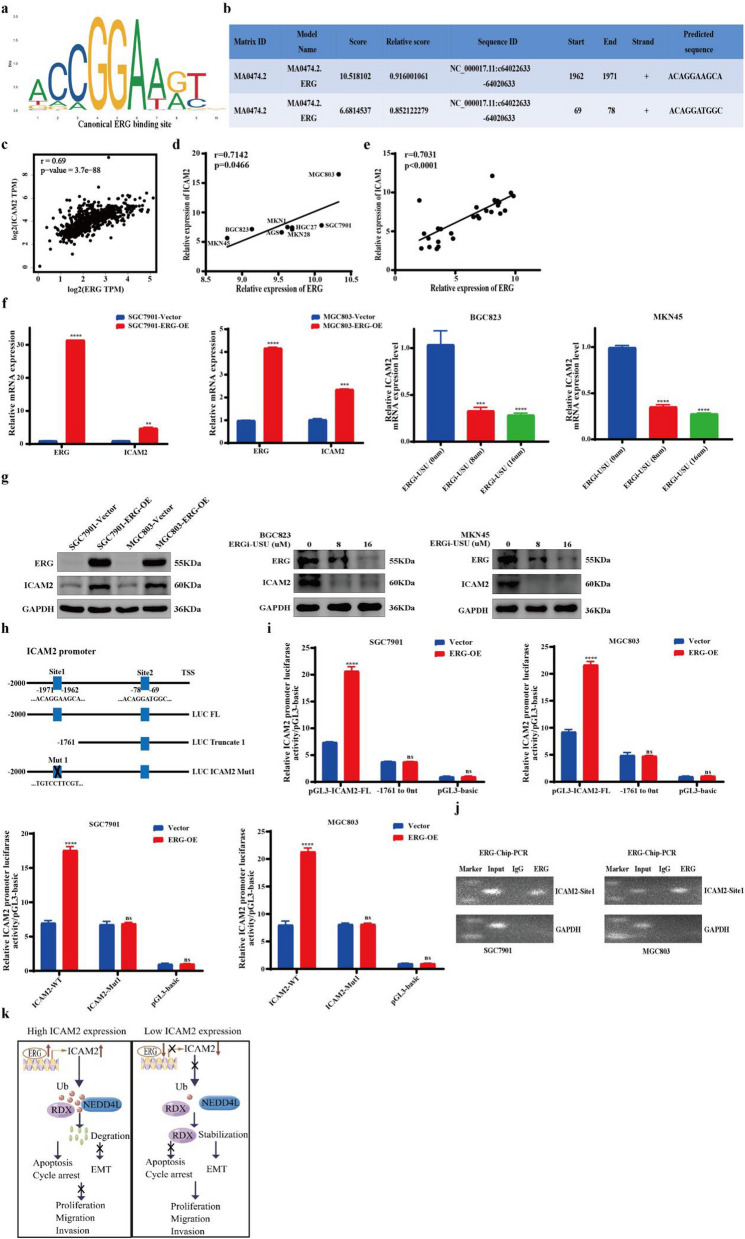
ERG-mediated upregulation of ICAM2 by binding to the promoter sequence of ICAM2. **a**, **b** JASPAR prediction of ERG-binding sites on the promoter region of ICAM2. **c–e** Correlation analysis between ERG and ICAM2 mRNA expression in GC cells and clinical GC tissue samples. **f**, **g** RT-PCR and Western blot analysis of ERG and ICAM2 expression in GC cells with ERG overexpression or inhibition. **h** ERG-binding elements (sites 1–2) on the ICAM2 promoter region. **i** The specific binding site 1 of ERG in the ICAM2 promoter region was identified through the dual-luciferase reporter assay via the truncation and mutation of the binding sites. **j** ChIP assay revealed ERG enrichment at the ICAM2 promoter site 1 in GC cells. **k** Specific mechanistic diagram of the ERG/ICAM2/RDX axis

To investigate the upstream regulatory mechanisms of ICAM2 in GC, we searched the JASPAR and TRRUST databases to identify transcription factors and putative transcription factor-binding sites in the ICAM2 promoter (2000 bp upstream of the transcriptional start site) (Fig. 7a, b). Results showed that ERG was a potential upstream transcriptional regulator of ICAM2. Furthermore, we explored the correlation between ERG and ICAM2 mRNA expression in GC tissues using an online database GEPIA. Results indicated that ERG strongly and positively correlated with ICAM2 expression in GC tissues (Fig. 7c). Moreover, this positive correlation was also detected in GC cells and tissues (Fig. 7d, e). To investigate whether ERG was functionally involved in the regulation of ICAM2 expression, we detected ICAM2 expression following ERG overexpression in SGC7901 and MGC803 cell lines and found that ERG overexpression upregulated ICAM2 expression levels (Fig. 7f). We then detected ICAM2 expression after ERG inhibition in BGC823 and MKN45 cell lines and found that ERG inhibition downregulated ICAM2 expression levels (Fig. 7f). Western blot results were consistent with changes in mRNA levels (Fig. 7g). In light of the above findings, we hypothesized that ERG can directly bind to the promoter region of ICAM2 and regulate ICAM2 transcription and expression. Thus, we explored the mechanism of ERG-induced ICAM2 gene transcription. Luciferase reporter assay was performed to investigate the ERG transcriptional regulation using the ICAM2 luciferase plasmid (Fig. 7h). It was found that ERG overexpression increased the luciferase activity of pGL3-ICAM2-FL (Fig. 7i). However, ERG overexpression had no significant effect on luciferase activity in pGL3-ICAM2-Truncated 1 (Fig. 7i). As expected, when binding site 1 was mutated, ERG overexpression caused a loss of luciferase activity (Fig. 7i). Subsequently, we designed one pair of ChIP–PCR primers based on site 1 sequences. The direct binding of ERG to the predicted site 1 on the ICAM2 promoter region was confirmed with ChIP (Fig. 7j). Taken together, these results demonstrated that ERG could bind to site 1 of the ICAM2 promoter region to induce ICAM2 transcription.

## Discussion

GC is ranked as the third most common cause of cancer-related deaths in China [28]. Although considerable progress has been made in the refinement of diagnostic techniques and therapies, most GC cases are diagnosed at an advanced stage, hence have a poor 5-year survival rate [29]. Therefore, multidisciplinary treatments, including systemic chemotherapy, radiotherapy, surgery, immunotherapy, and targeted therapy, are recommended. Among them, molecularly targeted treatments are emerging as a promising strategy with good outcomes [30, 31]. However, the clinical benefits of targeted drugs are limited by the development of drug resistance, unregulated expression levels of molecular targets, and low response rates. Therefore, there is a need to urgently identify novel therapeutic targets for GC treatment. ICAM2, a transmembrane glycoprotein of the immunoglobulin superfamily, regulates immune responses and contributes to the occurrence of some cancers [32]. Recent studies have reported that ICAM2 is deregulated in multiple human cancers [33]. However, whether ICAM2 plays a role in GC development remains unclear. Through analysis of the TCGA database, we observed a downregulation of ICAM2 expression in GC tissues compared to normal tissues. Furthermore, the mRNA expression of ICAM2 was found to be significantly reduced in GC cell lines compared to normal gastric mucosal epithelial cell lines. Consistent with results from in vitro experiments and those from the TCGA database, ICAM2 protein levels were lower in GC tissues compared to adjacent non-tumor tissues. Moreover, we observed a significant correlation between ICAM2 protein expression and clinical factors including clinical stage, T stage, distant metastasis, and vessel invasion in GC patients. These findings suggest that ICAM2 may influence GC proliferation and metastasis. Multivariate Cox regression analysis showed that downregulation of ICAM2 expression was an independent indicator of poor prognosis of GC patients. Subsequently, we established human GC cell line with ICAM2 overexpression and knockdown to clarify the role of ICAM2 in GC. Consequently, the results indicated that ICAM2 overexpression suppressed the proliferation, migration, invasion, and metastasis of GC cells both in vitro and in vivo. Conversely, ICAM2 knockdown yielded the opposite effects. These data suggested that ICAM2 has tumor-suppressive effects and can inhibit GC pathogenesis.

GC progression is a complex process involving multiple molecular interactions between tumor suppressor genes and oncogenes. Investigating the intricate regulatory mechanisms that underlie GC progression is crucial for developing innovative therapeutic approaches for GC patients. In the past several decades, research has indicated that motility and metastasis are regulated by membrane-cytoskeletal linker proteins which mediate interactions between cytoplasmic domains of membrane proteins and actin [34]. One study found that the cytoplasmic domain of ICAM2 interacts with the cytoskeletal linker protein α-actinin; this interaction promotes the non-metastatic phenotype of ICAM2 in neuroblastoma cells [15]. Considering that ICAM2 interacts with other proteins, we explored the proteins interacting with ICAM2 to improve our understanding of the mechanism by which ICAM2 inhibits GC development. We targeted RDX because it is a multifunctional membrane-cytoskeletal crosslinker protein. RDX can connect the cytoskeleton to the cell membrane via the FERM domain and is responsible for various cellular functions including proliferation, invasion, and adhesion [35–37]. Abnormal expression of RDX contributes to the development of several diseases and it may also promote tumor occurrence and progression by modulating the signaling pathways relevant to tumorigenesis [38]. For example, Valderrama F found that silencing RDX by siRNA inhibited the migration of PC3 prostate cancer cells and increased the cell area and cell–cell contacts mediated by adherent junctions. It also transformed their morphology to be similar to that of epithelial cells [21]. Hua D et al. revealed that miR-31 improved the migration and motility of glioma cells by specifically targeting RDX [39]. Building on this knowledge, we investigated the relationship between RDX and ICAM2. Our findings showed that ICAM2 overexpression significantly reduced the protein expression of RDX, without affecting its mRNA expression. This suggests that ICAM2 affects the post-transcriptional regulation of RDX. Moreover, we observed that the half-life of the RDX protein was markedly shortened in ICAM2-overexpressing cells treated with CHX, whereas accumulation of the RDX protein was increased in ICAM2-overexpressing cells treated with MG132. These results strongly indicate that ICAM2 promotes RDX protein degradation via the proteasome pathway. Previous studies have shown that ubiquitination is a crucial post-translational modification of the FERM domain. For instance, Cbl, an E3 ubiquitin ligase, interacts with and specifically ubiquitinates the JAK2 FERM and kinase domains via the Cbl TKB domain [40]. Similar to JAK2 protein structure, RDX contains an extended domain of the FERM protein at its N-terminus. These clues demonstrate that the regulation of RDX protein in GC cells may be driven by the ubiquitin-mediated proteasomal degradation pathway. This study shows that ICAM2 mediates the ubiquitination and degradation of RDX by interacting with RDX. Analysis of the UbiBrowser database implied that RDX ubiquitination was influenced by the four main E3 ubiquitin ligases. As a key tumor suppressor in human cancer, NEDD4L regulates the ubiquitin-mediated degradation of oncoproteins. Interestingly, we found that NEDD4L knockdown significantly attenuated RDX ubiquitination and increased RDX protein levels in GC cells overexpressing ICAM2. This indicates that ICAM2 promotes RDX ubiquitination and degradation in a NEDD4L-dependent manner. Protein interactions determine the molecular and cellular mechanisms that modulate pathogenic mechanisms, resulting in the onset and progression of diseases [41]. Herein, we uncovered that ICAM2 regulated the proliferation, migration, and invasion of GC cells by downregulating RDX expression because the ectopic expression of RDX abolished the inhibition of GC cell proliferation, migration, and invasion by ICAM2. These data indicate that ICAM2 interacts with RDX and promotes the ubiquitination-driven degradation of RDX in a NEDD4L-dependent manner to inhibit GC pathogenesis. Thus, identification of specific molecular interactions between ICAM2 and RDX may reveal important ideas for developing effective antitumor agents aimed at preventing or delaying metastasis of GC cells.

To elucidate the upstream regulatory mechanism of ICAM2, we explored the transcription factor that modulated ICAM2 expression. Through extensive bioanalysis, we observed a potential correlation between ERG and ICAM2 upregulation and ERG appeared to recognize two putative response elements located in the promoter region of ICAM2. Further analyses demonstrated that ERG, a transcription factor, could bind to site 1 of the ICAM2 promoter region to promote ICAM2 transcription, which potentiated the cancer-inhibitory effect of ICAM2. Studies have identified that ERG exerts oncogenic functions in some cancers [42], and it has recently been reported to be a therapeutic target in prostate cancer due to its critical role in prostate cancer development. However, the role of ERG in GC is currently unclear [43]. Although it can stimulate the transcription of oncogenes, the effects of ERG may be context-dependent [44]. These unprecedented findings suggest that strategies targeting ERG for cancer intervention, particularly in GC, should be carefully designed.

In summary, this study demonstrates the tumor-suppressive role of ICAM2 and its correlation with RDX in GC. ICAM2 inhibits GC cell proliferation and metastasis by ubiquitinating and degrading RDX in a NEDD4L-dependent manner to block GC progression. The findings unveil a previously unidentified axis, i.e., ERG-ICAM2-RDX, which modulates GC progression. Targeting this axis represents a promising and innovative therapeutic strategy for GC treatment.

## Conclusion

In conclusion, this study found that ICAM2 was downregulated in GC and this was positively correlated with advanced T stage, distant metastasis, advanced clinical stage, vessel invasion, and poor prognosis. In vitro and in vivo experiments revealed that ICAM2 significantly inhibited the growth and metastasis of GC cells. Mechanistically, ICAM2 was positively regulated by ERG resulting in inhibition of the proliferation and motility of GC cells by promoting NEDD4L-mediated ubiquitination and degradation of RDX. Taken together, these findings suggest that ICAM2 is a potential diagnostic marker and therapeutic target for GC patients.

### Supplementary Information

**Additional file 1. ICAM2 plays tumour‐suppressive roles in GC cell.** (a) The efficiency ICAM2 overexpression or knockdown were confirmed by RT-PCR. (b) Flow cytometry analysis of the effect of ICAM2 overexpression or knockdown on the cell cycle progression of GC cells. (c) Flow cytometry results showing the effect of ICAM2 overexpression or knockdown on the apoptosis of GC cells. (d) Representative image of the wound healing assays.

**Additional file 2. The predicted E3 ubiquitin ligases of RDX.** (a) UbiBrowser was used to predict the E3 ubiquitin ligases of the RDX. (b) RT-PCR was performed to verify the knockdown efficiency of the main E3 ubiquitin ligases in GC cells.

**Additional file 3.** **Restoration of RDX reverses the antitumor effect of ICAM2 overexpression in GC**. (a) The efficiency of RDX overexpression was confirmed by RT-PCR. (b) Representative image of the wound healing assays. (c) The apoptosis of ICAM2-overexpressing cells treated with RDX was confirmed by flow cytometry. (d) Transfection efficiency after overexpression of RDX in GC cells was determined by western blot.

## Data Availability

In the article or supplementary material, all data are provided.

## Abbreviations

GC: Gastric cancer
ICAM2: Intercellular adhesion molecule 2
qRT-PCR: Quantitative real-time polymerase chain reaction
ERG: Erythroblast transformation-specific-related gene
RDX: Radixin
NEDD4L: NEDD4 Like E3 Ubiquitin Protein Ligase
TMAs: Tissue microarrays
Co-IP: Co-immunoprecipitation
IHC: Immunohistochemistry
CHX: Cycloheximide
ChIP: Chromatin Immunoprecipitation;
GES-1: Normal gastric mucosal epithelial cells
ns: Not significant
EMT: Epithelial-to-mesenchymal transition

## References

[1] Sung H, Ferlay J, Siegel RL, Laversanne M, Soerjomataram I, Jemal A (2021). Global cancer statistics 2020: GLOBOCAN estimates of incidence and mortality worldwide for 36 cancers in 185 Countries. CA Cancer J Clin.

[2] Yamamoto Y, Yoshida N, Yano T, Horimatsu T, Uedo N, Kawata N (2022). Assessment of outcomes from 1-year surveillance after detection of early gastric cancer among patients at high risk in Japan. JAMA Netw Open.

[3] Kwon M, Kim G, Kim R, Kim KT, Kim ST, Smith S (2022). Phase II study of ceralasertib (AZD6738) in combination with durvalumab in patients with advanced gastric cancer. J Immunother Cancer.

[4] Arai J, Aoki T, Sato M, Niikura R, Suzuki N, Ishibashi R (2022). Machine learning-based personalized prediction of gastric cancer incidence using the endoscopic and histologic findings at the initial endoscopy. Gastrointest Endosc.

[5] Huang MT, Larbi KY, Scheiermann C, Woodfin A, Gerwin N, Haskard DO (2006). ICAM-2 mediates neutrophil transmigration in vivo: evidence for stimulus specificity and a role in PECAM-1-independent transmigration. Blood.

[6] Haghayegh Jahromi N, Marchetti L, Moalli F, Duc D, Basso C, Tardent H (2020). Intercellular adhesion molecule-1 (ICAM-1) and ICAM-2 differentially contribute to peripheral activation and CNS entry of autoaggressive Th1 and Th17 cells in experimental autoimmune encephalomyelitis. Front Immunol.

[7] Zaretsky I, Atrakchi O, Mazor RD, Stoler-Barak L, Biram A, Feigelson SW (2017). ICAMs support B cell interactions with T follicular helper cells and promote clonal selection. J Exp Med.

[8] Sager HB, Dutta P, Dahlman JE, Hulsmans M, Courties G, Sun Y (2016). RNAi targeting multiple cell adhesion molecules reduces immune cell recruitment and vascular inflammation after myocardial infarction. Sci Transl Med.

[9] Halai K, Whiteford J, Ma B, Nourshargh S, Woodfin A (2014). ICAM-2 facilitates luminal interactions between neutrophils and endothelial cells in vivo. J Cell Sci.

[10] Hiraoka N, Yamazaki-Itoh R, Ino Y, Mizuguchi Y, Yamada T, Hirohashi S (2011). CXCL17 and ICAM2 are associated with a potential anti-tumor immune response in early intraepithelial stages of human pancreatic carcinogenesis. Gastroenterology.

[11] Tanaka H, Yashiro M, Sunami T, Sakate Y, Kosaka K, Hirakawa K (2004). ICAM-2 gene therapy for peritoneal dissemination of scirrhous gastric carcinoma. Clin Cancer Res.

[12] Ishigami T, Uzawa K, Fushimi K, Saito K, Kato Y, Nakashima D (2008). Inhibition of ICAM2 induces radiosensitization in oral squamous cell carcinoma cells. Br J Cancer.

[13] Huang MT, Mason JC, Birdsey GM, Amsellem V, Gerwin N, Haskard DO (2005). Endothelial intercellular adhesion molecule (ICAM)-2 regulates angiogenesis. Blood.

[14] Feduska JM, Garcia PL, Brennan SB, Bu S, Council LN, Yoon KJ (2013). N-glycosylation of ICAM-2 is required for ICAM-2-mediated complete suppression of metastatic potential of SK-N-AS neuroblastoma cells. BMC Cancer.

[15] Feduska JM, Aller SG, Garcia PL, Cramer SL, Council LN, van Waardenburg RC (2015). ICAM-2 confers a non-metastatic phenotype in neuroblastoma cells by interaction with α-actinin. Oncogene.

[16] Sasaki Y, Tamura M, Takeda K, Ogi K, Nakagaki T, Koyama R (2016). Identification and characterization of the intercellular adhesion molecule-2 gene as a novel p53 target. Oncotarget.

[17] Yoon KJ, Phelps DA, Bush RA, Remack JS, Billups CA, Khoury JD (2008). ICAM-2 expression mediates a membrane-actin link, confers a nonmetastatic phenotype and reflects favorable tumor stage or histology in neuroblastoma. PLoS ONE.

[18] Wang X, Li N, Han A, Wang Y, Lin Z, Yang Y (2020). Ezrin promotes hepatocellular carcinoma progression by modulating glycolytic reprogramming. Cancer Sci.

[19] Clucas J, Valderrama F (2014). ERM proteins in cancer progression. J Cell Sci.

[20] Parameswaran N, Gupta N (2013). Re-defining ERM function in lymphocyte activation and migration. Immunol Rev.

[21] Valderrama F, Thevapala S, Ridley AJ (2012). Radixin regulates cell migration and cell-cell adhesion through Rac1. J Cell Sci.

[22] Zheng B, Liang L, Huang S, Zha R, Liu L, Jia D (2012). MicroRNA-409 suppresses tumour cell invasion and metastasis by directly targeting radixin in gastric cancers. Oncogene.

[23] Chen SD, Song MM, Zhong ZQ, Li N, Wang PL, Cheng S (2012). Knockdown of radixin by RNA interference suppresses the growth of human pancreatic cancer cells in vitro and in vivo. Asian Pac J Cancer Prev.

[24] Chen SD, Song MM, Zhong ZQ, Li N, Wang PL, Cheng S (2016). Knockdown of radixin suppresses gastric cancer metastasis in vitro by Up-regulation of E-cadherin via NF-κB/Snail pathway. Cell Physiol Biochem.

[25] Tsai MM, Wang CS, Tsai CY, Chen CY, Chi HC, Tseng YH (2017). MicroRNA-196a/-196b promote cell metastasis via negative regulation of radixin in human gastric cancer. Cancer Lett.

[26] Li T, Zhou J, Jiang Y, Zhao Y, Huang J, Li W (2022). The novel protein ADAMTS16 promotes gastric carcinogenesis by targeting IFI27 through the NF-κb signaling pathway. Int J Mol Sci.

[27] Jiang Y, Yu X, Zhao Y, Huang J, Li T, Chen H (2021). ADAMTS19 suppresses cell migration and invasion by targeting S100A16 via the NF-κB pathway in human gastric cancer. Biomolecules.

[28] Lin Y, Zheng Y, Wang HL, Wu J (2021). Global patterns and trends in gastric cancer incidence rates (1988–2012) and predictions to 2030. Gastroenterology.

[29] Deng L, Groman A, Jiang C, Perimbeti S, Gabriel E, Kukar M (2021). Association of preoperative chemosensitivity with postoperative survival in patients with resected gastric adenocarcinoma. JAMA Netw Open.

[30] Wang FH, Zhang XT, Li YF, Tang L, Qu XJ, Ying JE (2021). The chinese society of clinical oncology (CSCO): clinical guidelines for the diagnosis and treatment of gastric cancer, 2021. Cancer Commun.

[31] Shah MA, Kennedy EB, Alarcon-Rozas AE, Alcindor T, Bartley AN, Malowany AB (2023). Immunotherapy and targeted therapy for advanced gastroesophageal cancer ASCO guideline. J Clin Oncol.

[32] Amsellem V, Dryden NH, Martinelli R, Gavins F, Almagro LO, Birdsey GM (2014). ICAM-2 regulates vascular permeability and N-cadherin localization through ezrin-radixin-moesin (ERM) proteins and Rac-1 signalling. Cell Commun Signal.

[33] Zhang N, Liu X, Wu J, Li X, Wang Q, Chen G (2022). Serum proteomics screening intercellular adhesion molecule-2 improves intermediate-risk stratification in acute myeloid leukemia. Ther Adv Hematol.

[34] Barik GK, Sahay O, Paul D, Santra MK (2022). Ezrin gone rogue in cancer progression and metastasis: an enticing therapeutic target. Biochim Biophys Acta Rev Cancer.

[35] Jiang QH, Wang AX, Chen Y (2014). Radixin enhances colon cancer cell invasion by increasing MMP-7 production via Rac1-ERK pathway. ScientificWorldJournal.

[36] Qin JJ, Wang JM, Du J, Zeng C, Han W, Li ZD (2014). Radixin knockdown by RNA interference suppresses human glioblastoma cell growth in vitro and in vivo. Asian Pac J Cancer Prev.

[37] Ou R, Zhu L, Zhao L, Li W, Tao F, Lu Y (2019). HPV16 E7-induced upregulation of KDM2A promotes cervical cancer progression by regulating miR-132-radixin pathway. J Cell Physiol.

[38] Neisch AL, Fehon RG (2011). Ezrin, Radixin and Moesin: key regulators of membrane-cortexinteractions and signaling. Curr Opin Cell Biol.

[39] Hua D, Ding D, Han X, Zhang W, Zhao N, Foltz G (2012). Human miR-31 targets radixin and inhibits migration and invasion of glioma cells. Oncol Rep.

[40] Liu CS, Yang-Yen HF, Suen CS, Hwang MJ, Yen JJ (2017). Cbl-mediated K63-linked ubiquitination of JAK2 enhances JAK2 phosphorylation and signal transduction. Sci Rep.

[41] Kim M, Park J, Bouhaddou M, Kim K, Rojc A, Modak M (2021). A protein interaction landscape of breast cancer. Science.

[42] Gan W, Dai X, Lunardi A, Li Z, Inuzuka H, Liu P (2015). SPOP promotes ubiquitination and degradation of the ERG oncoprotein to suppress prostate cancer progression. Mol Cell.

[43] Rosen P, Sesterhenn IA, Brassell SA, McLeod DG, Srivastava S, Dobi A (2012). Clinical potential of the ERG oncoprotein in prostate cancer. Nat Rev Urol.

[44] Powell K, Semaan L, Conley-LaComb MK, Asangani I, Wu YM, Ginsburg KB (2015). ERG/AKR1C3/AR constitutes a feed-forward loop for AR signaling in prostate cancer cells. Clin Cancer Res.

