# Supplementary figures and images for: Intercellular adhesion molecule 2 as a novel prospective tumor suppressor induced by ERG promotes ubiquitination-mediated radixin degradation to inhibit gastric cancer tumorigenicity and metastasis
Source: J Transl Med. 2023 Sep 27;21:670. doi: 10.1186/s12967-023-04536-2 (PMC10536727; doi:10.1186/s12967-023-04536-2)

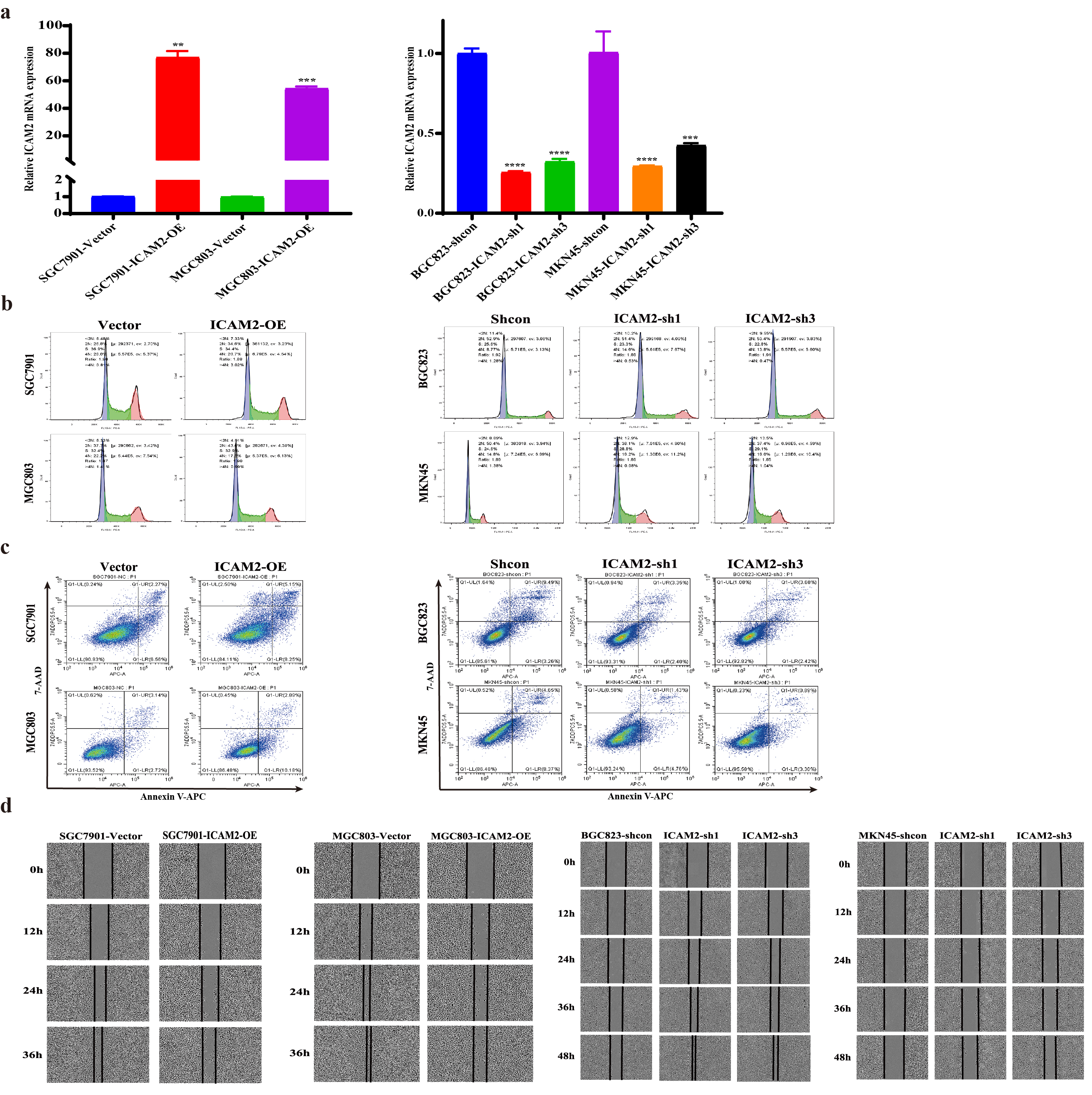

Supplement: Supplementary file 1 — Additional file 1. ICAM2 plays tumour‐suppressive roles in GC cell. (a) The efficiency ICAM2 overexpression or knockdown were confirmed by RT-PCR. (b) Flow cytometry analysis of the effect of ICAM2 overexpression or knockdown on the cell cycle progression of GC cells. (c) Flow cytometry results showing the effect of ICAM2 overexpression or knockdown on the apoptosis of GC cells. (d) Representative image of the wound healing assays. [file 12967_2023_4536_MOESM1_ESM.jpg]

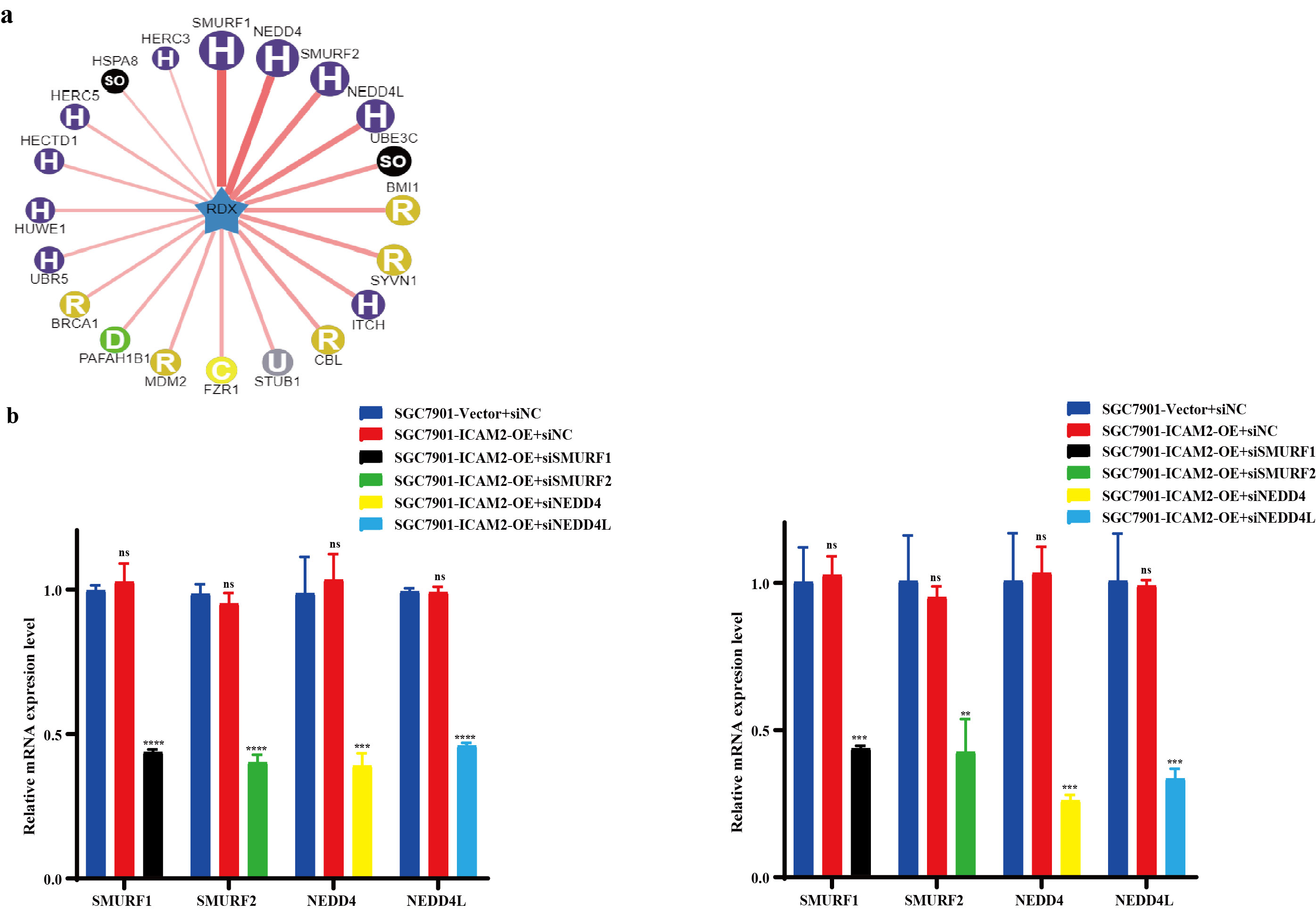

Supplement: Supplementary file 2 — Additional file 2. The predicted E3 ubiquitin ligases of RDX. (a) UbiBrowser was used to predict the E3 ubiquitin ligases of the RDX. (b) RT-PCR was performed to verify the knockdown efficiency of the main E3 ubiquitin ligases in GC cells. [file 12967_2023_4536_MOESM2_ESM.jpg]

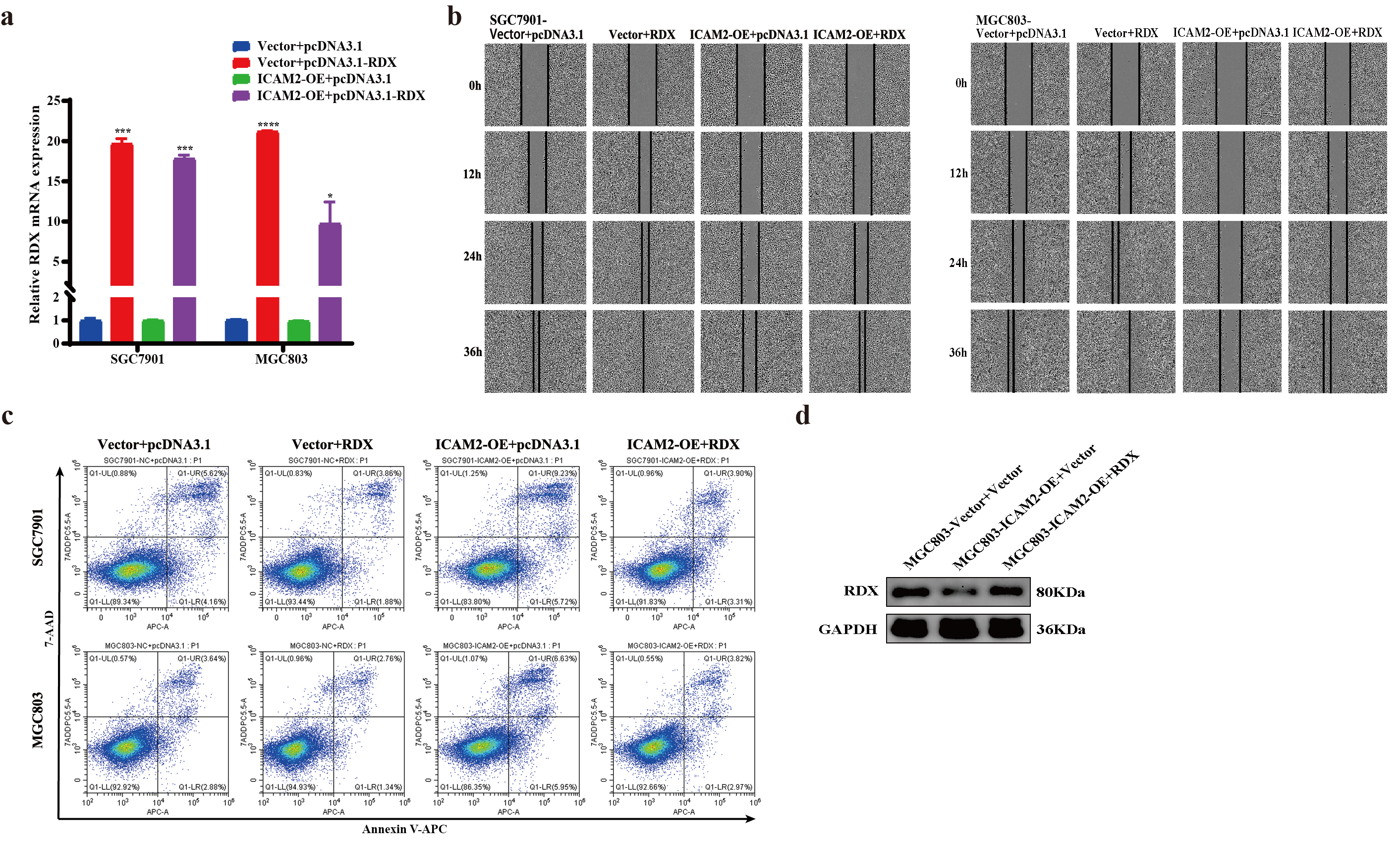

Supplement: Supplementary file 3 — Additional file 3. Restoration of RDX reverses the antitumor effect of ICAM2 overexpression in GC. (a) The efficiency of RDX overexpression was confirmed by RT-PCR. (b) Representative image of the wound healing assays. (c) The apoptosis of ICAM2-overexpressing cells treated with RDX was confirmed by flow cytometry. (d) Transfection efficiency after overexpression of RDX in GC cells was determined by western blot. [file 12967_2023_4536_MOESM3_ESM.jpg]
